# Sensitivities of a Standard Test Method for the Determination of the pHe of Bioethanol and Suggestions for Improvement

**DOI:** 10.3390/s101109982

**Published:** 2010-11-09

**Authors:** Richard J. C. Brown, Adam C. Keates, Paul J. Brewer

**Affiliations:** 1 Analytical Science Team, National Physical Laboratory, Teddington, Middlesex, TW110LW, UK; E-Mail: paul.brewer@npl.co.uk; 2 School of Chemistry, University of Southampton, Southampton SO17 1BJ, UK; E-Mail: adamkeates@gmail.com

**Keywords:** pHe, ethanol, biofuels, uncertainty, standard methods

## Abstract

An assessment of the sensitivities of the critical parameters in the ASTM D6423 documentary standard method for the measurement of pHe in (bio)ethanol has been undertaken. Repeatability of measurements made using the same glass electrode and reproducibility between different glass electrodes have been identified as the main contributors to the uncertainty of the values produced. Strategies to reduce the uncertainty of the measurement have been identified and tested. Both increasing the time after which the pHe measurement is made following immersion in the sample, and rinsing the glass electrode with ethanol prior to immersion in the sample, have been shown to be effective in reducing the uncertainty of the numerical value produced. However, it is acknowledged that the values produced using these modified approaches may not be directly compared with those obtained using the documentary ASTM method since pHe is defined operationally by the process used to measure it.

## 1. Introduction

Biofuels have the potential to replace or reduce the dependence on fossil fuels. The advantages biofuels have over fossil fuels are that they are potentially renewable and have the possibility to reduce overall greenhouse gas emissions. Many national and international legislation now specifies targets for reductions in future carbon emissions. In addition, some legislation requires the use of renewable energy sources for transportation (for example EC Directive 2009/28/EC [1]) also mentioning biofuels specifically (for example EC Directive 2009/30/EC [2]). As such bioethanol and biodiesel materials are being blended with fossil fuels to start the move to more environmentally sustainable energy frameworks. In particular the practice of blending bioethanol with petrol for use in motor vehicles is widespread—particularly in Brazil where blending is mandatory. As a result, quality-assuring bioethanol used for these applications is a key requirement of the trade, regulation and usage of the material. The measurement of ‘pHe’ is aimed at being a simple indicator of the corrosion potential of the bioethanol, and may be performed at most laboratories or as a field measurement with readily available equipment by technical staff—analogously to ‘pH’ for aqueous solutions [3]. As such, international specifications for bioethanol quality—EN 15376 [4] and ASTM D 4806 [5]—require the pHe value to be between 6.5 and 9.0 for anhydrous ethanol. The specifications call upon documentary standard test methods to perform these measurements: for example in Europe this is EN 15490 [6] and in the USA this is ASTM D6423 [7]. With the pHe requirement in Europe likely to be removed in the near future, the ASTM D6423 method for pHe determination has been brought into sharper focus as the main test method for pHe.

For a number of reasons the pHe of an ethanolic mixture bears no relation to the pH of an aqueous solution—whilst pHe is defined as the acid strength of ethanol, it is defined operationally by the apparatus and the method employed to make the measurement. Previous authors have referred to this juxtaposition as: “Apples are compared to oranges.” [3]. The reasons for this are partly because pHe measurement uses the same secondary measuring equipment (the glass pH electrode) and the same aqueous buffer solutions for calibration as its aqueous counterpart, but without a detailed understanding of the measurement being made [8], or with the traceability to the SI [9] that is available for aqueous pH measurements [10]. The result of this situation is that the numerical value produced by pHe measurements are dependent on the standard method used, and the type of glass electrode employed. To a certain extent, therefore, the presence of a detailed documentary standard method such as ASTM D6423 should provide measurements results with some limited stability and comparability (if not traceability or coherence) [11]. However a detailed investigation into the sensitivities of method to slight variations in key parameters such as measurement time, stirring rate, temperature, *etc.*, has never been undertaken. This paper presents data describing empirically determined sensitivities of the current ASTM D6423 method, additionally allowing a more robust estimate of uncertainty of the procedures to be made, and furthermore makes suggestions to improve the reproducibility and repeatability of the standard method which might be considered during any future revision. The authors are not aware of any existing relevant literature examining the ASTM D6423 method, although discussion of the basis and issues surrounding for pH determination in non-aqueous solution [12] is available [13] and guidelines for non-aqueous pH measurement [14] have been published [15], although in general these do not deal with mixtures of ethanol mass fraction close to 1 [16].

## 2. Experimental Section

All experimentation was conducted in a laboratory at 20 ± 2 °C. All chemicals used were of high-purity grade (Fisher), buffers were of high accuracy (Fisher) and solution were prepared gravimetrically throughout using deionised water (Millipore, MilliQ). Borosilicate glass vessels were used throughout. Prior to use these were thoroughly cleaned and washed and then rinsed with deionised water, before being filled with deionised water and left to stand for 48 hours to leach any remaining impurities adhered to the glass. The vessels were then rinsed again with deionised water and dried in an oven at 120 °C. When required, temperature control was exerted by placing the measurement vessels in a thermostatic water bath. Measurements were made based on the procedure described in the standard method ASTM D6423 [7]. The main variation from the method described in this standard was the use of a electrometer with high impedance (Keithley 2001) to record the voltage of the pH electrode in real time, rather than employing a pH meter which does not give a real time output and may also provide only time-averaged data. Additional small variations (as described at the relevant location in the text) were made to this method to allow the effect of different experimental parameters to be tested. For completeness the standard method employed is summarised below. The glass pH electrode used was the ORION Ross Sure-Flow combination electrode (ORION Cat. No. 8172BN). This was cleaned and re-hydrated before its first use and after the measurement of every ten samples by alternatively soaking several times in 1 M NaOH and 1 M H_2_SO_4_. The electrode was then rinsed and calibrated with pH 7.00 and pH 4.00 aqueous buffers, rinsing with deionised water between each solution. The electrode was then rinsed again and stored in the pH 7.00 buffer until use. About 50 mL of sample was placed in a 100 mL beaker and the solution was stirred at a rate such as to produce a vortex in the solution between 6 and 8 mm deep. The electrode was removed from the pH 7.00 buffer, rinsed with deionised water, and blotted to remove the excess solution. The electrode was then placed into the sample and the voltage (or pHe) reading taken after 30 ± 1 s. Ethanol samples with a nominal water content 0.02 g/g were measured during experimentation, unless otherwise stated.

## 3. Results and Discussion

**Figure 1.**
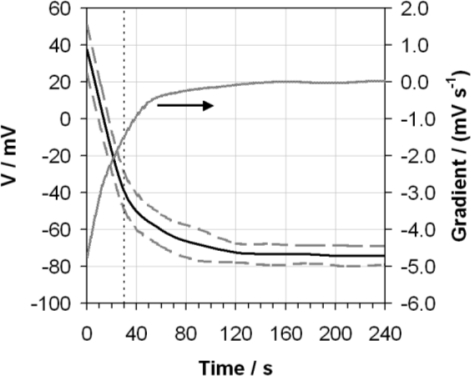
Average voltage (V) over ten measurements (black line) exhibited by the glass electrode as a function of time when measuring ethanol containing 0.02 g/g of water using the ASTM D6423 method. The standard deviation of this measurement set (dashed grey line) and the gradient of the average voltage response (grey line, right-hand axis) are also shown. The ASTM D6423 specified measurement time is indicated (vertical dotted line).

Figure 1 displays the average of 10 repeat measurements of an ethanol sample with a nominal water content 0.02 g/g. It is clear that upon starting the measurement the voltage exhibited by the glass electrode drops significantly. The rate of this decrease is highlighted by the plot of the gradient of this curve, also shown in Figure 1. After the defined measurement time of 30 s the voltage recorded was still changing at a rate of 1.5 mV s^−1^. After about 50 s the rate of voltage decrease slowed significantly, and by 120 s a approximate steady state response had been achieved. Figure 1 additionally shows the repeatability of the responses as the standard deviation over ten separate measurements using the same electrode.

**Figure 2.**
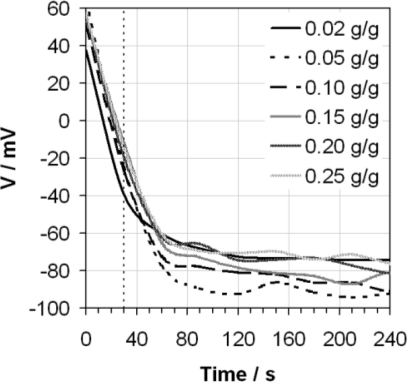
Voltage (V) exhibited by the glass electrode as a function of time when measuring ethanol containing varying mass fractions of water (as indicated by the legend) using the ASTM D6423 method. The ASTM D6423 specified measurement time is also indicated (vertical dotted line).

For comparison purposes, Figure 2 shows the average response for the same electrode in different compositions of water in ethanol mixtures. This shows that the shape of the measured response is broadly similar, but that the voltage recorded after 30 s may differ by up to 30 mV. This is to be expected given that changes in the composition of the mixture will result in changes in the autoprotolysis constant for the mixture [12], plus any additional effect there may be from small amounts of ionic content introduced following the addition of extra quantities of water.

**Figure 3.**
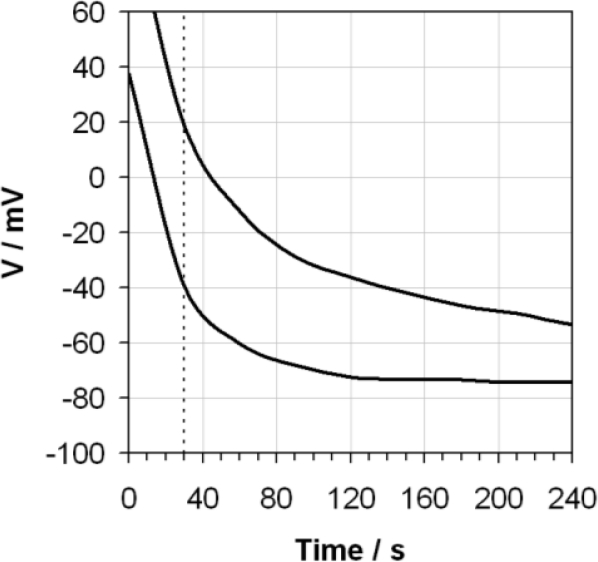
The average maximum and minimum voltages (V) exhibited by three different glass electrodes as a function of time when measuring ethanol containing 0.02 g/g of water using the ASTM D6423 method. The ASTM D6423 specified measurement time is also indicated (vertical dotted line).

Figure 3 shows the reproducibility limits of the average response from three different glass electrodes (all nominally identical), which clearly shows a significantly larger spread in data than was observed under repeatability conditions for the same electrode.

**Figure 4.**
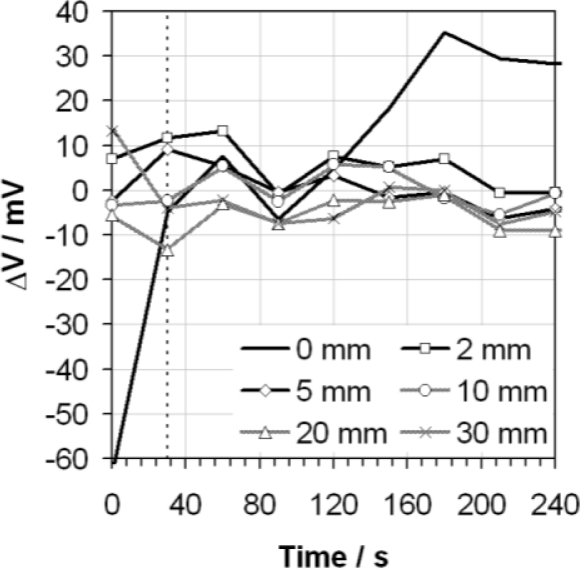
Differential voltage (ΔV) exhibited by the glass electrode as a function of time when measuring ethanol containing 0.02 g/g of water using the ASTM D6423 method, for a variety of vortex depths (as indicated in the legend), relative to that measured for a vortex depth of 7 mm. The ASTM D6423 specified measurement time is also indicated (vertical dotted line).

The sensitivity of the response to the depth of the vortex in the solution created by stirring during measurement is shown in Figure 4, plotted relative to the response obtained at the mid-point of the recommended range: 7 mm. It is clear that not stirring the sample at all causes very variable results. The results obtained when the mixture is stirred shows some variability in the time domain but, in general, slower stirring rates result in higher voltage responses and faster stirring rates result in lower voltage responses.

**Figure 5.**
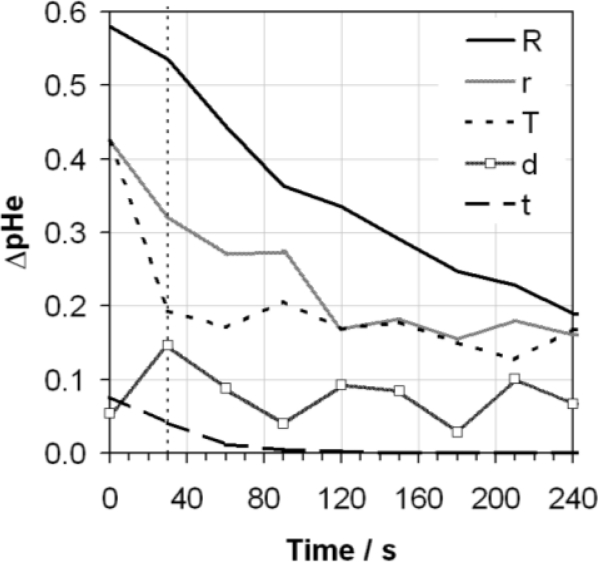
Absolute variability in pHe (ΔpHe) as a function of time cause by variability observed in the key parameters of: reproducibility (R, on the legend); repeatability (r); solution temperature (T); vortex depth (d); and measurement time (t), when using the ASTM D6423 method to measure ethanol containing 0.02 g/g of water. The ASTM D6423 specified measurement time is also indicated (vertical dotted line).

The responses obtained from the same electrode at different temperatures from 15 to 35 °C were also measured (not displayed graphically here). This allowed a sensitivity of measured voltage response to temperature to be calculated, as ΔV/ΔT, as a function of time. This quantity feeds into the uncertainty analysis described below. Following the studies described above the variability of the parameters investigated were converted in variability in measured pHe, using the calibration slope of the glass electrode in aqueous buffer determined during the measurement procedure. The results of this analysis are shown in Figure 5.

The reproducibility contribution has been calculated based on the range of values shown in Figure 3 divided by $\sqrt{3}$-treating this as a rectangular distribution. The repeatability contribution is simply the standard deviation of the response from the same electrode, as shown in Figure 1. The contribution from vortex depth has been calculated as the range of pHe values obtained using the allowable range of vortex depths of between 6 and 8 mm, detailed in the ASTM method. Temperature dependence has been assessed as the differences in pHe measured across the range of temperatures allowable by the ASTM of between 20 and 24 °C. Finally, the contribution from measurement time takes into account the range of pHe values which would have been obtained over the allowable measurements times mentioned in the procedure—between 29 and 31 s—using the gradient of the voltage response determined in Figure 1. Assuming the remainder of the standard method is followed it is proposed that these components are the main contributors to the variability of the measurement.

**Figure 6.**
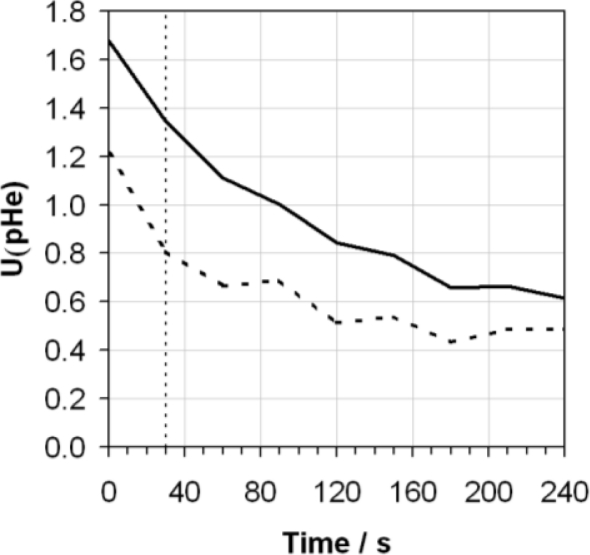
Expanded uncertainty [U(pHe)], assuming a coverage factor of k = 2, giving a level of confidence of approximately 95% calculated as a function of time by combining in quadrature the data displayed in Figure 5 including (solid line) and excluding (dashed line) the contribution of reproducibility between electrodes. The ASTM D6423 specified measurement time is also indicated (vertical dotted line).

It is noteworthy that the contributions from reproducibility and repeatability at the 95% confidence interval (assuming a coverage factor of k = 2) after 30 s as determined in Figure 5, of 0.90 and 0.64 pHe, respectively, are significantly greater than those values suggested in the ASTM method of 0.52 and 0.29 pHe. Whilst the data set used to produce the data in the ASTM standard may be significantly larger than presented here, this already provides an indication of the difficulty of making reproducible pHe measurements. If the data presented in Figure 5 is taken to represent individual contributions to the standard uncertainty as a function of time, these contributions may be combined in quadrature and expanded by a coverage factor of 2 to give an estimate of the overall expanded uncertainty of the measurement at the 95% confidence level. This has been performed in Figure 6. It can be seen that for the two cases considered, with and without the reproducibility component included, the predicted uncertainties are relatively large at lower measurement times, particularly at 30 s where the ASTM method suggests measurement, but drop significantly after longer times eventually levelling off at approximately 0.65 pHe for the uncertainty including reproducibility, and approximately 0.50 pHe for the uncertainty excluding reproducibility. These uncertainties are clearly quite large, and limit the usefulness of the data obtained from the pHe measurement.

The results presented above highlight the need to propose methodological improvements to reduce the uncertainty of the numerical value obtained from pHe measurements. Given that pHe is an operationally defined measurand, it is feasible to make suggestions to improve the measurement method itself, in order to improve the uncertainty of the numerical result determined. Changes to the method will, of course, cause the measured pHe values to change, and hence specifications for the range of pHe values allowed for compliance in bioethanol specifications would also have to change as a result—this is the price of dealing with operationally defined parameters. The largest contributions to the overall uncertainty come from repeatability and reproducibility and so efforts to reduce the overall uncertainty of measurements should initially concentrate in this area. It is clear that the magnitude of both these parameters decrease at longer measurement times. Hence a practical solution to decreasing measurement uncertainty is simply to increase the time after which the glass electrode is introduced into the sample that the measurement is taken. This has the advantage of the decreasing the uncertainty contribution of most of the parameters considered because, whilst still drifting slightly, the voltage reading is significantly more stable at longer timescales than it is at 30 s. At a measurement time of 2 min the uncertainties in the pHe value would be roughly half the value recorded at a measurement time of 30 s.

**Figure 7.**
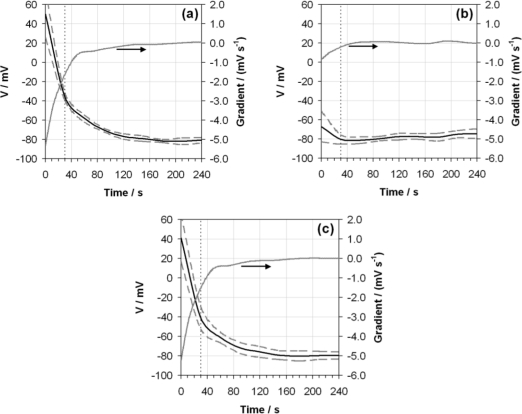
Average voltage (V) over ten measurements (black line) exhibited by the glass electrode as a function of time when measuring ethanol containing 0.02 g/g of water using the ASTM D6423 method with variations to encompass: **(a)** the use of buffers containing 10% ethanol; **(b)** washing the glass electrode with ethanol prior to measurement and; **(c)** using a electrode filling solution containing 10% ethanol. The standard deviation of this measurement set (dashed grey line) and the gradient of the average voltage response (grey line, right-hand axis) are also shown. The ASTM D6423 specified measurement time is indicated (vertical dotted line).

In terms of decreasing the contribution to the overall uncertainty from reproducibility, increasing measurement times is the only simple solution. Other considerations such as investigating alternative designs of electrode are difficult, costly and outside the scope of this study. Needless to say, for an operationally defined measurand as critically dependent on the measuring device as pHe, it is essential that measuring devices are produced to high quality and exacting specifications. However, other more radical mechanisms for decreasing the repeatability of the response of the same electrode are more experimentally accessible. Three different strategies have been employed to further improve the repeatability of measurement and reduce electrode drift as a function of time. Similar proposals to improve the quality and traceability of routine pHe measurements have previously been made [17]. The effect of these strategies is shown in Figure 7 (analogously to the presentation of data in Figure 1). In terms of changes to the ASTM method, these were:

- The use of buffer mixtures comprised of 90% aqueous buffer solution and 10% ethanol (by volume), including standing and rinsing the electrode in these mixtures prior to use (Figure 7(a)).
- Rinsing the glass electrode with ethanol instead of water prior to use (Figure 7(b)).
- Employing a glass electrode filling solution comprised of 90% aqueous 3 mol dm^−3^ KCl solution and 10% ethanol (by volume) (Figure 7(c)).

It is clear from these investigations that the use of the non-aqueous filling solution (Figure 7(c)) has very little effect on electrode response in terms of both voltage profile and repeatability—producing a plot almost identical to Figure 1. The use of non-aqueous buffers (Figure 7(a)) similarly produces little change in the voltage profile, but the standard deviation of response is improved by a factor of 2 across the time domain. The combined use of non-aqueous buffers and non-aqueous filling solution produced results very similar to those shown in Figure 7(a). However, rinsing the bulb with ethanol before making the pHe measurement (Figure 7(b)) produced dramatically different voltage profiles, showing very little drift over time, and reaching an approximate steady state by the defined measurement time of 30 s. In addition, the repeatability of the electrode response shows improvements of a factor of 2 across the time domain as compared to the data in Figure 1.

**Figure 8.**
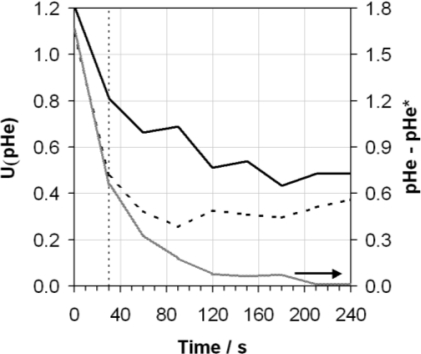
Expanded uncertainty [U(pHe)] (assuming a coverage factor of k = 2, giving a level of confidence of approximately 95%) calculated as a function of time by combining in quadrature the relevant individual components (excluding the contribution of reproducibility between electrodes) for the ASTM method (solid line) and the ASTM method but washing the glass electrode with ethanol prior to measurement (dashed line). The difference between the numerical values obtained using the ASTM method (pHe) and the ASTM method but washing the glass electrode with ethanol prior to measurement (pHe*) are also shown (grey line, right hand axis). The ASTM D6423 specified measurement time is indicated (vertical dotted line).

It is apparent from the results in Figure 7 that some initial dehydration of the glass electrode bulb prior to measurement of the sample solution avoids substantial voltage drift over time and additionally helps improve the repeatability of the response. Unfortunately it is not possible to prepare buffer mixtures of filling solution with very high ethanol contents owing to the lack of solubility of the buffer or salt in these mixed solvents—this is a well-known limitation of the use of buffers in ethanolic solutions for such applications. Hence the buffer solutions and filling solution containing only 10% ethanol by volume were not as successful in stabilising the electrode response as when the glass electrode was washed in pure ethanol prior to use. The uncertainty contributions (excluding reproducibility) to the measurement of pHe when washing the glass electrode in ethanol prior to use have been calculated, and an expanded uncertainty produced (analogously to Figure 6) are compared against the expanded uncertainty (excluding reproducibility) for the ASTM method without the use of the ethanol washing step, in Figure 8.

Figure 8 also gives the absolute difference in pHe measured using the two methods as a function of time. It is clear that the uncertainty of the results produced are uniformly lower as a result of using the ethanol wash before measurement, mainly as a consequence of the improved repeatability across the time domain. The difference between the pHe measured using the ASTM method and that using the ASTM method with the ethanol wash (referred to as pHe*) is initially very large but soon converges to within 0.3 pHe after 1 min, and less than 0.1 pHe after 2 min. This is because the pHe value starts high and drifts downwards quickly, asymptotically approaching the pHe* value which shows very little variability across the time domain, principally because much of the dehydration of the glass electrode will have already occurred during the rinsing step.

## 4. Conclusions

The sensitivities of the ASTM D6423 method for the measurement of pHe to variations in the most important parameters have been tested. It has been determined that the most important contributory factors to the variability, and therefore uncertainty, in the measured pHe value are the reproducibility between different electrodes and the repeatability of measurements made using the same electrode. The substantial voltage drift exhibited by the measurement during the first two minutes exacerbates this situation. Based on the data collected the uncertainty of the method has been estimated as a function of time. It is noted that this uncertainty decreases substantially with measurement time. Hence it has been suggested therefore that the existing method could be improved by extending the time at which the pHe reading is taken from 30 s to 2 min to allow more time for the system to equilibrate. This proposal limits the effect of lack of electrode reproducibility on the overall measurement, which is the most difficult parameter to mitigate without expensive investigations into new electrode designs.

Further investigations have assessed the effect of the use of non-aqueous buffers and non-aqueous electrode filling solutions on the performance of the method. These had little effect on the overall performance of the method, apart from an improvement in repeatability when using the non-aqueous buffers. In addition, the effect of an ethanol rinse prior to making the measurement was assessed. This had the result of dramatically reducing the drift at short timescales, presumably as a result of partially dehydrating the glass electrode prior to measurement. In addition there was an improvement in repeatability. Assessment of the uncertainty of this method showed that it produced values of pHe* of lower uncertainty than pHe measurements produced using the full ASTM method. In addition, after measurement time of approximately 2 min, the two techniques produced nominally identical numerical values. Hence it has been additionally suggested that the use of an ethanol wash prior to measurement may produce assessments of “pHe” with lower uncertainties.

Importantly, it has been recognized throughout that pHe is an operationally defined measurand and that any changes to the method used to measure it will alter the meaning, and most likely the numerical value, of the quantity itself. This has the additional effect that, were proposals to improve the pHe measurement to be adopted, they would require a concomitant change in published bioethanol specifications to alter the allowable range of “pHe” values accordingly.
